# Beneficial effects of a plant-fish oil, slow carbohydrate diet on cardio-metabolic health exceed the correcting effects of metformin-pioglitazone in diabetic pigs fed a fast-food diet

**DOI:** 10.1371/journal.pone.0257299

**Published:** 2021-10-20

**Authors:** Sietse J. Koopmans, Heleen M. M. van Beusekom, F. Josef van der Staay, Gisabeth Binnendijk, Marcel Hulst, Zlaw Mroz, Mariette T. Ackermans, Lambertus Benthem

**Affiliations:** 1 Wageningen Livestock Research, Wageningen University & Research, Wageningen, The Netherlands; 2 Experimental Cardiology, Erasmus Medical Center, Rotterdam, The Netherlands; 3 Department of Farm Animal Health, Veterinary Faculty, Utrecht University, Utrecht, The Netherlands; 4 Wageningen Bioveterinary Research, Lelystad, The Netherlands; 5 Department of Animal Science and Bioeconomy, University of Life Sciences, Lublin, Poland; 6 Endocrine Laboratory, Clinical Chemistry, Amsterdam UMC, Location AMC, Amsterdam, The Netherlands; 7 Inorbit Therapeutics AB, Mölndal, Gothenburg, Sweden; University of Cordoba, SPAIN

## Abstract

**Background:**

Lifestyle influences endocrine, metabolic and cardiovascular homeostasis. This study investigated the impact of diet and oral anti-diabetic medication on cardio-metabolic health in human-sized diabetic pigs.

**Methods:**

After a growing pre-phase from ~30 to ~69 kg during which domestic pigs were fed either a low fat, low sucrose diet (group A) or a fast food-type diet elevated in lard (15%) and sucrose (40%) (group B), the pigs were subdivided in 5 groups (n = 7–8 pigs per group). Group 1, normal pigs from group A on a low fat, low sugar (**L**) pig diet and group 2, normal pigs from group B on a high lard (25%), sucrose-fructose (40%), cholesterol (1%) fast food-type (**F**) diet. Diabetes (**D**) was induced in group B pigs by streptozotocin and group 3 received the **F** diet (**DF**), group 4 received the **F** diet with **A**nti-diabetic medication metformin (2 g.day^-1^)-pioglitazone (40 mg.day^-1^) (**DFA**) and group 5 switched to a **P**lant-**F**ish oil (25%), **S**lowly digestible **s**tarch (40%) diet (**DPFS**). The **F** and **PFS** diets were identical for fat, carbohydrate and protein content but only differed in fat and carbohydrate composition. The 5 pig groups were followed up for 7 weeks until reaching ~120 kg.

**Results:**

In normal pigs, the **F** diet predisposed to several abnormalities related to metabolic syndrome. Diabetes amplified the inflammatory and cardiometabolic abnormalities of the **F** diet, but both oral **FA** medication and the **PFS** diet partially corrected these abnormalities (mean±SEM) as follows: Fasting plasma TNF-ɑ (pg.ml^-1^) and NEFA (mmol.l^-1^) concentrations were high (p<0.02) in **DF** (193±55 and 0.79±0.16), intermediate in **DFA** (136±40 and 0.57±012) and low in **DPFS** pigs (107±31 and 0.48±0.19). Meal intolerance (response over fasting) for glucose and triglycerides (area under the curve, mmol.h^-1^) and for lactate (3-h postprandial, mmol.l^-1^) was high (p<0.03) in **DF** (489±131, 8.6±4.8 and 2.2±0.6), intermediate in **DFA** (276±145, 1.4±1.1 and 1.6±0.4) and low in **DPFS** (184±62, 0.7±1.8 and 0.1±0.1). Insulin-mediated glucose disposal (mg.kg^-1^.min^-1^) showed a numerical trend (p = NS): low in **DF** (6.9±2.2), intermediate in **DFA** (8.2±1.3) and high in **DPFS** pigs (10.4±2.7). Liver weight (g.kg^-1^ body weight) and liver triglyceride concentration (g.kg^-1^ liver) were high (p<0.001) in **DF** (23.8±2.0 and 69±14), intermediate in **DFA** (21.1±2.0 and 49±15) and low in **DPFS** pigs (16.4±0.7 and 13±2.0). Aorta fatty streaks were high (p<0.01) in **DF** (16.4±5.7%), intermediate in **DFA** (7.4±4.5%) and low in **DPFS** pigs (0.05±0.02%).

**Conclusion:**

This translational study using pigs with induced type 2 diabetes provides evidence that a change in nutritional life style from fast food to a plant-fish oil, slowly digestible starch diet can be more effective than sole anti-diabetic medication.

## Introduction

Medication such as metformin [1, 2] and/or pioglitazone [3, 4] is used to treat the pathophysiology of obese type 2 diabetes. Lifestyle, like diet and physical exercise, also plays an important role in both the prevention and treatment of obese type 2 diabetes mellitus [5, 6]. It becomes more and more clear that a change in lifestyle may be very effective, not only in the prevention, but also in the treatment of obese type 2 diabetes [7–9]. With respect to food intake, not only the amount of diet consumed but also the composition of the diet is important as a life-style factor to correct the pathophysiology of obese type 2 diabetes and to improve cardiometabolic health [10, 11]. Plant-fish oil, slowly digestible carbohydrate diets have been implicated with improved cardiometabolic homeostasis in obese and type 2 diabetic patients [12–15]. In general, plant-fish oil, slowly digestible carbohydrate diets are characterized by high monounsaturated fatty acid (MUFA) content, and fruit and vegetables, whole grains, fish, and nuts, and low in saturated fats (SFA) [16]. More specifically, dietary fat composition (percentage of MUFA, polyunsaturated fatty acids (PUFA) and SFA) has been shown to differentially affect cardiometabolic risk factors in metabolic syndrome patients [17, 18]. However, a standardized comparative study at the systemic, tissue and organ level which quantifies the underlying mechanisms related to cardiometabolic health in type 2 diabetic subjects on oral anti-diabetic medication or on a plant-fish oil, slowly digestible carbohydrate diet has, as yet, to our knowledge not been conducted. Such a long-term and invasive study is difficult to perform in diabetic patients as discussed previously [19] and therefore we have used a human-size large animal model resembling the pathophysiology of human type 2 diabetes. We have developed such a pig model for human diabetes mellitus type 2 expressing relevant cardiometabolic abnormalities [20–23]. These pigs are non-ketotic, anabolic and do not require insulin therapy [19, 20]. Pigs, like humans, are omnivores and as such the functionality of the gastrointestinal tract is comparable between pig and man. Therefore the pig is a useful animal model for the study of dietary components and oral drugs [24–26].

The present study compared the medium-term (7 weeks) impact of a fast food diet without or with oral anti-diabetic medication (combined administration of metformin-pioglitazone) and the impact of a plant-fish oil, slow carbohydrate diet on cardio-metabolic health in domestic diabetic pigs. Cardio-metabolic health of the pigs was characterized by pre- and postprandial glycemia, lipidemia and insulinemia, by insulin sensitivity, blood pressure, circulating pro-inflammatory markers, glucose transporters in jejunal mucosa, insulin staining area of the pancreas and by muscle, liver and aorta lipid deposits.

## Materials and methods

Experimental protocols describing the management, surgical procedures, and animal care were reviewed and approved by the ASG-Lelystad Animal Care and Use Committee (Lelystad, The Netherlands). The performed research is in compliance with the ARRIVE guidelines on animal research [27].

### Animals, housing and diets

Sixty male crossbred (Yorkshire x Landrace, T-line) 11-week old pigs (barrows), weighing approximately 30 kg (supplier: Bastiaanse, Espel, The Netherlands) were group housed in 8 pens. The pigs in two pens (group A) were fed a commercial pig diet (5% crude fat, 16% crude protein, 41% starch and sugars, 20% non-starch polysaccharides, 6% ash and 12% water; Startbrok, Agrifirm, Meppel, The Netherlands) as a low fat, low sucrose recommended diet for 30 kg pigs and the pigs in six pens (group B) were fed a high fat (15% lard), high sucrose (40%) diet for 30 kg pigs (growing pre-phase, **Table 1).** The high fat, high sucrose diet was designed to meet the nutrient requirements of 30 kg pigs according to the Dutch feed evaluation system for growing pigs (Centraal Veevoeder Bureau 2011. CVB table pigs. Product Board Animal Feed, The Hague, The Netherlands [28]). All pigs were fed ad libitum and had free access to water. At a weight of approximately 69 kg, 38 out of 60 pigs (7 pigs of the low fat, low sucrose diet group A and 31 pigs of the high fat, high sucrose diet group B) were selected, balanced on body weight and housed individually in metabolism cages (1.15 x 1.35 m). The ambient room temperature was 20°C. All pigs were adapted to the light-dark cycle–lights being on from 05:00 to 22:00 h–and a regime with two meals a day staying at the same type of food (low fat, low sucrose diet or high fat, high sucrose diet). The pigs had ad libitum access to this food between 06:00 and 07:00 and between 15:00 and 16:00 h. Food consumption was registered daily by weighing the provided meal (twice a day) and weighing back the remaining food once a day. After the growing pre-phase, the pigs were fed the experimental diets. The composition of the low fat-low sugar (L) pig diet, the fast-food (F) diet and the plant-fish oil, slow carbohydrate (PFS) diet is presented in **Table 1** and the experimental diets were designed to meet the nutrient requirements of 70–100 kg pigs according to the Dutch feed evaluation system for growing pigs (Centraal Veevoeder Bureau 2011. CVB table pigs. Product Board Animal Feed, The Hague, The Netherlands, [28]). Gross energy, crude protein and fat contents and contents of monounsaturated fatty acids (MUFA), polyunsaturated fatty acids (PUFA) and saturated fatty acids (SFA) are shown. All diets, except for the commercial pig diet as used in the growing pre-phase, were tailor-made by Research Diet Services, Wijk bij Duurstede, The Netherlands. Pigs were weighed once every 14 days.

**Table 1:** Composition of the experimental diets.

Raw material	Growing pre phase		Experimental phase		
Diet	Low fat-low sucrose commercial pig diet	High fat-high sucrose	Low fat-low sugar	Fast food	Plant-fish oil, slow carbohydrate diet
Wheat middlings		150.0			
Soya beans, extracted (cf<50 g/kg)		200.0		164.5	164.5
Potato protein (ash < 10 g/kg)		60.0	50.0	50.0	50.0
Maize gluten meal		12.4			
Animal fat (lard)		150.0		250.0	
Barley			396.2		
Wheat			500.0		
Wheat gluten meal			8.7	106.0	106.0
Sucrose		400.0		200.0	
Fructose				200.0	
Pea starch					400.0
Soya oil			17.3		
Trisun sunflower oil					55.9
Canola oil					142.5
Corn oil					45.0
Fish oil					6.6
Limestone		7.7	13.0	9.6	9.6
Mono calcium phosphate		10.6	6.9	10.8	10.8
NaCl		3.9	4.0	4.7	4.7
Mineral/vitamin-Premix			2.0	2.0	2.0
Choline Chloride 500		0.3			
Mineral/vitamin premix M2763		4.0			
L-Lysine HCl		0.3	1.9	2.4	2.4
DL-Methionine		0.8			
Cholesterol (extra)				10.0	
Total (g/kg)		1000.0	1000.0	1010.0	1000.0
Crude fat (g/kg)	50	160	40	260	260
MUFA (g/kg)	12	72	11	119	156
PUFA (g/kg)	30	24	23	36	79
SFA (g/kg)	8	64	6	104	24
Crude protein (g/kg)	160	160	170	170	170
Gross Energy (GE; MJ/kg)	16.3	21.3	17.3	23.5	23.5

The percent of each ingredient contributing to the total amount of diet (= 100%) is listed. The Pre phase commercial pig diet is a low sucrose-low fat diet (see text Material and Methods).

^1^ This vitamin and trace mineral premix contained per kg diet: vit. A (retinol)– 1750 IU; vit. D_3_ (cholecalciferol)– 200 IU; vit. E (tocopherol)– 11 IU; vit. K_1_ (phylloquinone)– 0.5 mg; vit. B_1_ (thiamin)– 1.0 mg; vit. B_2_ (riboflavin)– 4 mg; d-pantothenic acid– 9 mg; niacin (vit. B_3_, nicotinic acid)– 12.5 mg (available); biotin (vit. H)– 50 μg; vit. B_12_ (cyanocobalamin)– 15 μg; folic acid (folacin)– 0.3 mg; vit. B_6_ (pyridoxin)– 1.5 mg; choline– 400 mg; Fe– 80 mg; Zn– 54 mg; Mn– 30 mg; Co– 0.15 mg; I– 0.14 mg; Se– 0.25 mg; antioxidants (E310,320,321)– 50 mg; with maize starch as carrier.

### Timeline

**Fig 1:**
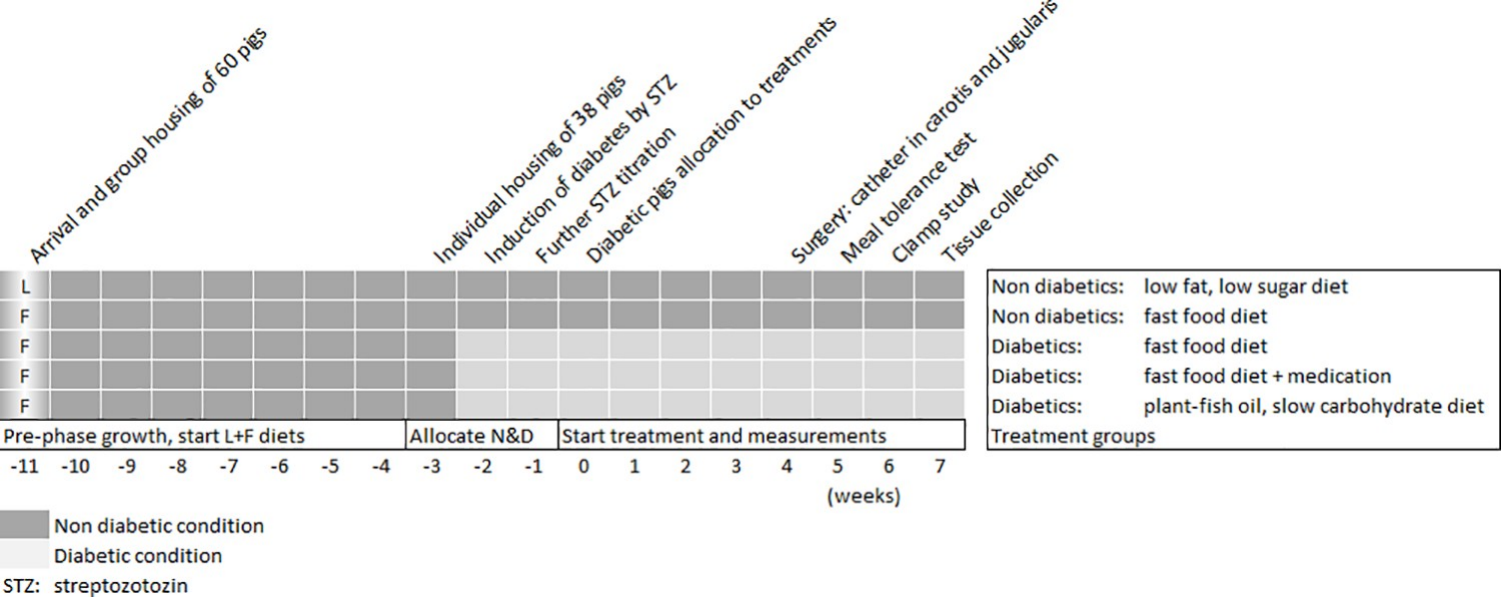
Timeline of the study in normal (non-diabetic) and diabetic pigs, showing the growing pre-phase with the two diets to select groups with balanced growth; the induction of D in a subset of animals and the allocation to the treatment groups. At week -3, 38 out of 60 pigs with comparable body weight were balanced and selected for the experimental treatments and placed in metabolic cages. At week 0, start of the experimental treatments. L = low fat, low sugar diet, F = fast-food, D&N = diabetic and non-diabetic pigs.

The timeline of the study is represented schematically in **Fig 1**. In short, after the growing pre-phase (lasting 11 weeks) from ~30 to ~69 kg during which normal pigs were fed either a low fat, low sucrose diet (group A) or a fast food-type diet elevated in saturated fat (15% lard) and sucrose (40%) (group B), the pigs were subdivided in 5 groups (n = 7–8 pigs per group) at week 0. Group 1 (n = 7), normal pigs from group A on a low fat, low sugar (**L**) diet and group 2 (n = 7), normal pigs from group B on a high saturated fat (25% lard), sucrose-fructose (40%), cholesterol (1%) fast food-type (**F**) diet. Diabetes (**D**) was induced in group B pigs by streptozotocin and group 3 (n = 8) received the **F** diet (**DF**), group 4 (n = 8) received the **F** diet with **A**nti-diabetic medication metformin (2 g.day^-1^)-pioglitazone (40 mg.day^-1^) (**DFA**) and group 5 (n = 7) switched to a high unsaturated fat (25% **P**lant-**F**ish oil), **S**lowly degradable carbohydrate (40% pea starch) diet (**PFS**). The 5 treatment groups were followed-up for 7 weeks comprising the following techniques and measurements: 1) daily food intake, 2) 2-weekly body weight, 3) instrumentation with permanent catheters in the jugular vein and carotid artery (week 4), 4) a meal tolerance test with intra-arterial blood pressure recording (week 5), 5) a hyperinsulinemic euglycemic clamp study with 6,6-^2^H_2_-glucose infusion (week 6), and 6) euthanasia by intravenous injection of barbiturate (administered via the jugular catheter) for tissue collection at week 7.

### Induction of diabetes

In week -3 (see **Fig 1**), type 2-like diabetes was induced after overnight fasting by injecting 24 pigs of the high fat, high sucrose diet group B with citrate-buffered streptozotocin (STZ) (80 mg.kg^-1^, Pharmacia & Upjohn Company, Kalamazoo, Michigan, USA). Prior injection, STZ was dissolved in saline (1 g STZ per 10 mL saline) and administered by slow infusion over a 30 minute time period through an ear vein catheter (Becton Dickinson, Secalon Seldy, 16 G, polyurethane, Franklin Lakes, New Jersey, USA). During the first day after STZ treatment, all pigs had free access to food in order to avoid hypoglycemia. Over the next days, the food was offered still ad libitum but twice daily (06:00–7:00, 15:00–16:00) to evaluate the food intake capacity in relation to 24h urinary glucose excretion of diabetic pigs. The daily urinary glucose excretion guided additional STZ injections (resp. on 3 and 6 days after the first dose) to equalize the severity of diabetes among pigs. One week after the last STZ injection (week -1, see **Fig 1**) the Diabetic pigs were assigned to one of three different treatments (Fast food without (DF) or with Anti-diabetic medication (DFA) or plant-fish oil, slow carbohydrate diet (DPFS) as follows: during week -1 (see **Fig 1**), i.e. the week after the last STZ infusion (days 6–12 after the final dose of STZ), the severity of diabetes was estimated using urinary glucose excretion (g.day^-1^) as an index. The pigs were rank-ordered depending on the severity of diabetes and assigned to one of three experimental diabetic groups using random matched assignment. Mean STZ dose (mg.kg^-1^ bodyweight) and mean urinary glucose excretion (g.day^-1^) were 124±10, 132±7, 129±5 mg/kg and 492±70, 469±80, 477±61 g.day^-1^ in DF (n = 8), DFA (n = 8), DPFS (n = 7) pigs respectively. The treatments started at week 0 (see **Fig 1**).

### Surgeries

Surgery was preceded by an overnight fast (water available ad libitum). The day after surgery, pigs were fed 50% of pre-surgically consumed food, followed by re-establishing preoperative food intake the following days. Pigs were anaesthetised by intramuscular injection of 2 mg azaperone.kg^-1^ body mass (Stressnil; Janssen, Tilburg, The Netherlands) followed by an intravenous injection of 15 mg Nesdonal.kg^-1^ body mass (Rhone Merieux, Lyon, France). Pigs were intubated and general anesthesia was maintained by inhalation of 2% Sevoflurane (Abbott) combined with 40% oxygen and nitrous oxide. The pigs were equipped with polyethylene catheters (Tygon, i.d. 1.02 mm, o.d. 1.78 mm, length 1 m; Norton, Akron, Ohio, USA) four weeks after the start of administration of the experimental diets. The jugular vein catheter was inserted and advanced until the tip of the catheter reached the antrum and the carotid artery catheter was advanced until the tip reached the aortic arch. The catheters were fixed firmly at the place of insertion and were tunneled subcutaneously to the back of the pig and exteriorized between the shoulder blades. The catheters were filled and sealed with physiological saline containing 50 IU heparin and 150.000 IU penicillin (Procpen; AUV, Cuijk, The Netherlands) per ml and kept in and protected by a backpack which was glued to the skin of the pig’s back. During surgery the pig was given an intramuscular injection of antibiotic (300.000 IU procaine penicillin G, Depocilline, Mycofarm Nederland B.V., De Bilt, The Netherlands) and anodyne (50 mg flunixine, Finadyne, Schering-Plough N.V./S.A., Brussels, Belgium).

During the one-week recovery period after surgery, the pigs were habituated to blood sampling and infusion procedures. The jugular vein catheter was used for infusion of fluids during the study. The carotid artery catheter was used for blood sampling and the measurement of blood pressure and heart rate. During the blood sampling procedure, the catheters were flushed and filled with physiological saline containing 5 IU.ml^-1^ heparin. From experience we know that this sampling procedure does not affect plasma NEFA concentrations. After the one-week postsurgical recovery and habituation period, the pigs were treated with streptozotocin to induce diabetes.

### Anti-diabetes medication

The medication group received twice per day 20 mg Pioglitazone + 1 g Metformin in a moistened food ball (50 g food) before each meal. The dose was based on clinical guidelines [29, 30]. In the USA and the EU, the recommended combination dose for humans is 15 mg for Pioglitazone and 850 mg for Metformin, administered once or twice daily in patients with type 2 diabetes in whom glycemia is inadequately controlled by metformin alone. Metformin monotherapy has been shown to be effective in streptozotocin-induced diabetes before in pigs by us and in rodents by others [20, 31].

### Meal tolerance test

In week 5 after the start of the treatments, blood was sampled in each pig repeatedly before, during and after the administration of a standardized morning meal (-30, -15, 15, 30, 45, 60, 90, 120, 180, 240, 300, 360, 420 and 480 minutes). The size of the standardized morning meal corresponded with 2.5-fold maintenance requirements for gross energy (GE) as established in a normal pig. This was equivalent to a level of 522 kJ GE kg^-1^ BW^0.75^ per meal and is sufficient to ensure moderate growth in normal pigs [32]. Per sampling time point, approximately 5 mL blood was sampled. The responses of insulin, glucose and triglycerides were assessed in the plasma samples -15, 30, 60, 120, 240, 360 and 480 minutes from the start of the meal.

### Blood pressure and heart rate

Subsequent to the meal tolerance test, the blood pressure and heart rate were determined five hours post-prandially on a Digital Electromanometer (Type 330, Hugo Sachs Elektronik KG, March-Hugstetten, Germany). In resting position, blood pressures and heart rates were recorded in the pigs in 30-sec intervals for a 10-minute period.

### Hyperinsulinemic euglycemic clamps and 6,6-2H-glucose infusion

In week 6, after overnight fasting, preceding the clamp, two baseline EDTA and heparin blood samples were collected for later analyses of various parameters including insulin, glucose and background glucose enrichment.

Subsequently, after baseline samples, a prime (4.8 mg.kg^-1^)-continuous (0.08 mg.kg^-1^.min^-1^) infusion of 6,6-^2^H_2_-glucose was administered for 150 min. After an equilibration time, blood samples were taken at 110, 120, 130, 140 and 150 min for determination of glucose, 6,6-^2^H_2_-glucose enrichment (to estimate fasting hepatic glucose production) and insulin. Subsequently, insulin was administrated as prime (34 mU.kg^-1^)-continuous (2 mU.kg^-1^.min^-1^) infusion for 6 hours. A variable infusion of a 33% D-glucose solution was started to maintain plasma glucose at euglycemia (~5 mmol.L^-1^). Steady state calculations were carried out during the last 40 minutes of the clamp (t = 320, 330, 340, 350 and 360 min) and the coefficients of variation for the insulin and glucose concentrations, and for the infusion rate of glucose were determined (see Results section). During the last 3 hours of the insulin clamp, a prime (4.8 mg.kg^-1^)-continuous (0.08 mg.kg^-1^.min^-1^) infusion of 6,6-^2^H_2_-glucose was superimposed to estimate 1) insulin stimulated whole body glucose uptake (rate of disappearance = Rd) and 2) insulin-inhibited hepatic glucose production, as described before [20]. For this purpose, blood samples were taken at t 320, 330, 340, 350 and 360 min of the clamp.

Infusates: Insulin (Actrapid MC, porcine monocomponent, Novo, Copenhagen, Denmark), 6,6-^**2**^H_**2**_-glucose (Cambridge Isotope Laboratories, Inc, MA, USA) and D-glucose (Merck, Darmstadt, Germany) were prepared as sterile solutions and passed through a 0.22 μm Millipore filter into sterile containers before use. Insulin was diluted in a saline solution containing pig plasma (final plasma concentration was 3%) in order to avoid sticking of insulin to the plastic containers and tubings. 6,6-^**2**^H_**2**_-glucose was dissolved in a saline solution and **d**-glucose was dissolved in aqua dest.

### Tissue collection

In week 7, after overnight fasting, blood samples were collected in heparin and EDTA tubes 15 minutes before a standardized meal (522 kJ GE kg^**-1**^ BW^**0.75**^) and 180 minutes after the meal just prior to tissue collection. Pigs were euthanised by intravenous injection of an overdose barbiturate administered via the jugular vein catheter at immediate exsanguination. All samples were collected on ice. The intestines were taken out, the jejunum was opened, and the mucosa of 10 cm of jejunum was scraped off with a glass slide. Mucosal scrapings were snap frozen in liquid nitrogen and kept at -80 ^**o**^C until further use [33]. In addition, the spleen, thymus, internal fat depots and liver were dissected and weighed. A biopsy was taken from the musculus iliopsoasis and from the ventral liver lobe. Samples of these tissues were snap-frozen in liquid nitrogen and stored at -80°C until further use. Trigylceride concentrations were determined in the muscle and liver samples with the same kit as used for plasma after saponification with an alkaline alcohol solution according to Eggstein and Kreuz [34].

### Plasma, urine and tissue analyses

Blood samples collected in heparinised (150 USP. U. Lithium Heparin, 10 mL, Venoject, Terumo, Leuven, Belgium) or EDTA (ethylenediaminetetraacetic acid, (0.47 mol/L EDTA, 10 mL, Venoject, Terumo, Leuven, Belgium) tubes were immediately chilled in melting ice, and centrifuged at 4°C for 10 minutes at 3000 rpm. Plasma aliquots were stored at -80°C for later analyses. Urine was quantitatively collected per 24 hours in buckets containing 0.5 grams Halamid-d (sodium-p-toluenesulfonchloramide, Akzo Nobel Chemicals, Amersfoort, The Netherlands) to prevent microbial breakdown of glucose. Urine samples were stored at -20°C for later glucose analyses. Abdominal aorta was fixed in a 4% paraformaldehyde solution.

Plasma samples for determination of 6,6-^2^H_2_-glucose enrichment were analyzed as described previously [19, 20]. In short, glucose was extracted from plasma, derivatised, and injected into a gas chromatograph/mass spectrometer system (HP 6890 series GC system and 5973 Mass Selective Detector, Palo Alto, CA, USA). Separation was achieved on a J&W scientific DB 17 capillary column (30 m x 0.25 mm x 0.25 μm; Agilent Technologies Nederland BV, Amstelveen, The Netherlands). Isotopic enrichment was calculated as tracer-to-tracer ratio after subtracting the isotopic enrichment of a background plasma sample. An aliquot of the 6,6-^2^H_2_-glucose infusate was analyzed for the isotope concentration to calculate the actual infusion rate for each infusion.

Plasma glucose was analyzed with the Glucose liquiUV mono kit (Human, Wiesbaden, Germany), plasma non-esterified fatty acids were analyzed with the WAKO kit (Neuss, Germany) and plasma triglycerides with a kit from Human (Wiesbaden, Germany). Total, LDL and HDL cholesterol concentrations in plasma were determined with liquicolor kits (Human, Wiesbaden, Germany). VLDL cholesterol was calculated as total cholesterol minus LDL and HDL cholesterol. Plasma insulin concentration was measured using a Delfia assay (test kit by Perkin Elmer Life Sciences Trust by Wallac Oy, Turku, Finland). This specific pig insulin assay was validated using pig insulin standards, as indicated before [20]. Plasma cortisol was measured with the Count-A-Count Cortisol kit (DPC, Los Angeles, USA) and fructosamine by a kit from Spinreact (Sant Esteve De Bas, Spain). Tumor necrosis factor alpha (TNF-α) was analyzed with the CRP-hs kit from Human, Wiesbaden, Germany.

Plasma β-Hydroxy butyric acid was analyzed enzymatically using an auto analyzer (ADVIA 1650, Bayer Corp., Tarrytown, NY) and lactate concentrations were analyzed enzymatically using an auto analyzer (ABL, AML, Radiometer, Copenhagen, Denmark).

Plasma alanineaminotransferase (ALAT) and aspartateaminotransferase (ASAT) were analyzed on an Advia 1800 chemistry system (Siemens Health care Diagnostics, Tarrytown, NY, USA). Urinary ketones (acetoacetic acid) were determined in fresh urine by a reagent strip test (Ketostix, Bayer Diagnostics, Mijdrecht, The Netherlands).

Triglyceride concentrations in muscle and liver samples were determined with the same kit as used for the plasma samples, after saponification with an alkaline alcohol solution as described previously [34].

Aortic fatty streaks, as a marker of early atherosclerosis [35, 36], AHA class 2 lesion [37], were quantified en-face following an Oil-Red-O lipid stain in segments of the abdominal aorta ranging from the bifurcation of the renal arteries to the bifurcation of the iliac arteries. The stained aortas were then photographed and analyzed with a microscopy image analysis system (Clemex technologies Inc., Quebec, Canada) as ratio of stained area to total area. Cryosections were cut from representative segments and stained by Oil-Red-O to confirm the lipid rich nature of atheromatous plaque.

### Gene expression of the intestinal glucose transporters (SLC2A1, SLC2A2 and SLC2A5)

The gene expression of the glucose transporters (SLC2A1, SLC2A2 and SLC2A5) was determined by PCR. *Isolation of total RNA*: Approximately 1 gram of frozen tissue collected per pig was homogenized directly in 10 ml TRIzol^®^ reagent (GibcoBRL). After homogenization, insoluble material was removed by centrifugation at 12,000 × G for 10 min at 4°C. Further extraction of RNA from these homogenates was performed according to instructions of the manufacturer of TRIzol^®^ reagent. The crude RNA pellet obtained from this isolation procedure was dissolved in 1 ml RNase-free water, and precipitated with 0.25 ml of isopropanol and 0.25 ml of 0.8 M sodium citrate/1.2 M NaCl to remove proteoglycan and polysaccharide contamination. After centrifugation at 12,000 × g for 10 min at room temperature RNA pellets were washed with 75% (v/v) ethanol and dissolved in RNase-free water. Subsequently, the RNA was treated with DNase, extracted with phenol-chloroform, and precipitated with ethanol. RNA pellets were washed with 75% (v/v) ethanol, dissolved in RNase-free water, and stored at -70°C until further use. The integrity of the RNA was checked by analyzing 0.5 μg on a 0.9% (w/v) agarose gel. A part of the isolated RNA was used to prepare RNA pools for microarray analysis. For the tissues under study, equal amounts of RNA isolated from all pigs within each diet group were mixed and stored at -70°C until further use [33, 38].

#### Real time PCR

The expression levels of SLC2A5 (alias GLUT5 or GluT5), SLC2A1 (alias GLUT1 or SGlutT1) and SLC2A2 (alias GLUT2 or GluT2) mRNA in individual RNA samples were determined by real time PCR. For each sample 200 ng of RNA was reverse transcribed in a standard RT reaction using Superscript II reverse transcriptase (Invitrogen) and pd(N)_6_ primers. The sequences of the forward and reverse primers used to quantify SLC2A5, SLC2A1 and SLC2A2 cDNA fragments in RT reactions are depicted in **Table 2.** Cybergreen was used as label for detection of amplified cDNA fragments in a LightCycler real-time PCR machine (Roche Diagnostics). The relative amounts of SLC2A5, SLC2A1 and SLC2A2 mRNA in all RT reactions was calculated by extrapolation of the cycle crossing point on a standard curve prepared from dilutions of an RNA reference sample with relative high concentrations of SLC2A5, SLC2A1 and SLC2A2. The concentration of 18S rRNA was determined in the same RT reactions by real time PCR [39] and used to normalize the relative amounts of SLC2A5, SLC2A1 and SLC2A2 mRNA in all individual samples. The expression level of 18S rRNA showed no essential differences among all jejunal RNA samples (n = 26); mean expression±SD.; 57±14.ng.μl^-1^.

**Table 2:** Sequences of the forward and reverse primers to quantify glucose transporters cDNA fragments in RT reactions.

GENE (alias)	primers (5’-3’)	reference sequence acc. Number	position (length nt.)
SLC2A5 (GLUT5 or GluT5)	forward: TTGGTGAATCACTTAGGCAG	XM_021095282.1	793–1040 (247)
	reverse: ATGCCAACGGTGATGAAGAG		
SLC2A1 (GLUT1 or SGlutT1)	forward: GGAATTCCATGCTGATGATG	XM_021096908.1	534–845 (311)
	reverse: CCGGGATGAAGATGACGCTC		
SLC2A2 (GLUT2 or GluT2)	forward: GAAAGGGAAGAAGCATCAAG	NM_001097417.1	836–1388 (552)
	reverse: GTCCACAGAAGTCCGCAATG		

### Statistical analysis

Statistical evaluations were performed per time point by analysis of variance (two-way ANOVA) [40, 41] with the factor diabetic or non-diabetic and with the factor low fat, low sugar (L) diet, fast food (F) diet, fast food diet plus oral anti-diabetic medication (FA) and plant-fish oil, slow carbohydrate (PFS) diet. Subsequently, when ANOVA indicated a significant difference among groups, post-hoc analyses for between or within group comparisons were done with the two-sided unpaired or paired Student’s t-test of Genstat 19.1 (VSN International, Hemel Hempstead, UK.) [42]. Two-way ANOVA was used for data calculated as area under the curve (AUC) and two-way ANOVA for repeated measurements was used for data with repeated time points. The major readouts of the present study were the data collected during the post-prandial blood sampling period (week 5), the clamp study (week 6) and the section (week 7). In addition, analyses were performed with the repeated measures factor time point. Depending on the measure analyzed this may be minutes or hours after administration of a compound, or weeks from the start of administration of the experimental diets without or with medication.

Effects with associated *p*-values <0.05 were considered as statistically significant. If not indicated otherwise, means per time point and treatment condition ± standard error of the mean (SEM) are depicted in figures and listed in tables. *Means s*haring the same superscript A,B,C,D are not significantly different from each other.

## Results

### Food intake and body weight

**Fig 2:**
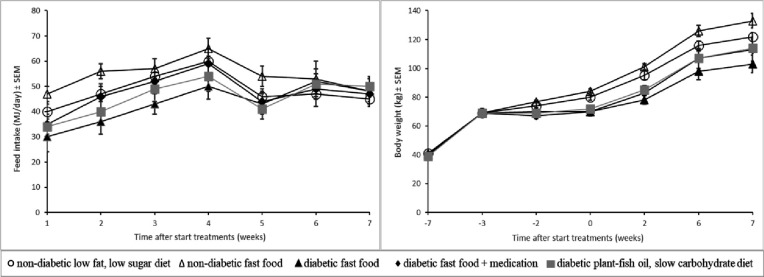
Daily food consumption and body weight gain (means±SEM) in five groups of normal (non-diabetic) and diabetic pigs fed a low fat, low sugar diet, fast food diet with or without medication or a plant-fish oil, slow carbohydrate diet.

Ad libitum food intake (MJ.day^-1^) from starting the treatments is shown in **Fig 2**. From weeks 1 through 4, food intake increased and average food intake of the NF pigs was high (56 MJ.day^-1^) compared to DF pigs (40 MJ.day^-1^), p<0.02. Food intake of the other groups was in-between. From weeks 4 through 7, average food intake was similar among groups and the plateauing of food intake coincided with surgery of the pigs in week 4 and episodes of restricted food intake during surgery and testing. Body weight gain is shown in **Fig 2**. Up to week -3, prior to STZ treatment, the body weights of the 5 experimental groups were similar and from week -2 onwards body weights of the experimental groups started to diverge. At the end of the study (week 7 at necropsy), significant (p<0.05) differences existed between experimental groups with high body weight in NF pigs (133±5 kg) and low body weight in DF pigs (103±7 kg).

### Meal tolerance test

**Fig 3:**
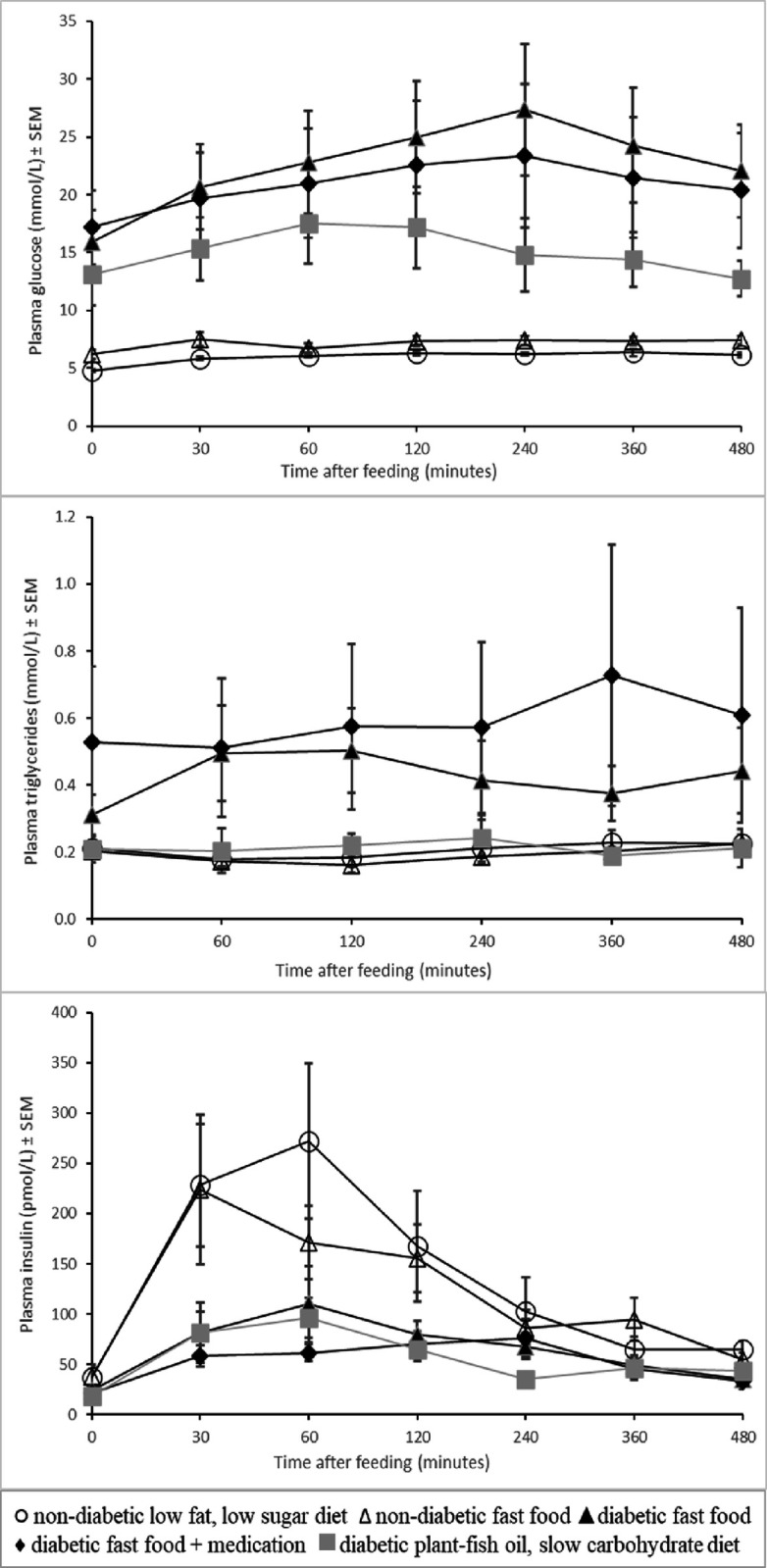
Mean glucose, triglyceride and insulin concentrations in the meal tolerance tests (means±SEM) in five groups of normal (non-diabetic) and diabetic pigs fed a low fat, low sugar diet, fast food diet with or without medication or a plant-fish oil, slow carbohydrate diet.

**Fig 4:**
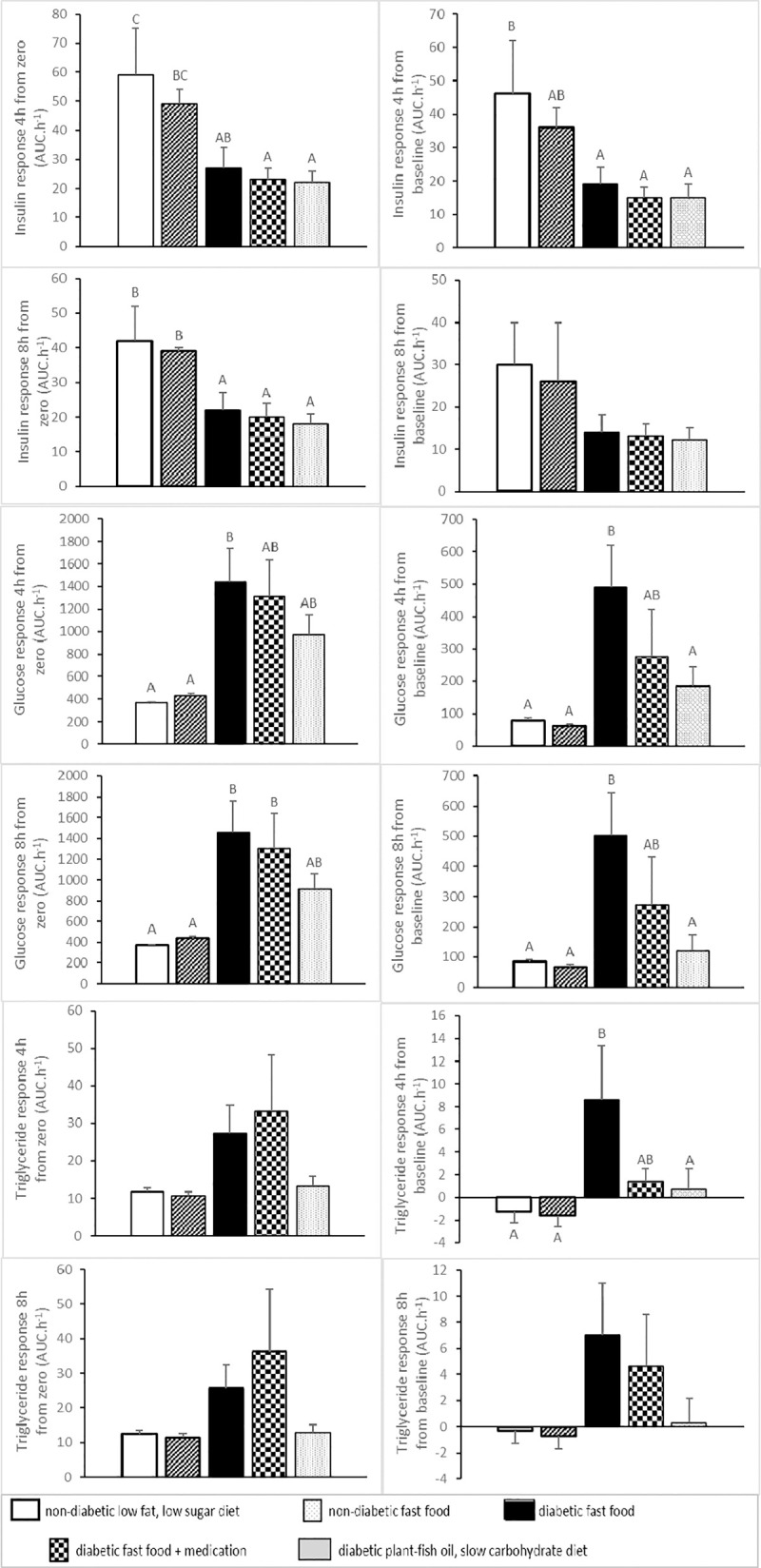
Meal-induced plasma glucose, triglyceride and insulin responses (means±SEM) in five groups of normal (non-diabetic) and diabetic pigs fed a low fat, low sugar diet, fast food diet with or without medication or a plant-fish oil, slow carbohydrate diet. **Areas under the curves (AUCs) are calculated from zero and baseline value (response), covering the period 0 to 4 hours and 0 to 8 hours postprandial (from start of the morning meal) and expressed in AUC·h**^**-1**^. Means sharing the same superscript A,B,C are not significantly different from each other.

Postprandial plasma glucose, triglyceride and insulin profiles are shown in **Figs 3** and **4** (meal tolerance test, week 5). Glucose response from baseline (area under the curve for 4 and 8 hours postprandially) was low (p<0.03) in normal pigs and DPFS pigs, intermediate in DFA pigs and high in DF pigs. Triglyceride response from baseline (area under the curve for 4 hours postprandially) was low (p<0.03) in normal pigs and DPFS pigs, intermediate in DFA pigs and high in DF pigs. Insulin response (area under the curve for 4 and 8 hours postprandially) was high (p<0.05) in normal pigs compared to D pigs.

### Blood pressure and heart rate

Blood pressure and heart rate, measured 5 hours after initiation of the meal tolerance test, are shown in **Table 3**. Mean arterial pressure, systolic and diastolic pressure and heart rate were similar among pig groups.

**Table 3:** Arterial blood pressures and heart rates.

	Normal–Low fat, low sugar diet	Normal–Fast food	Diabetic–Fast food	Diabetic–Fast food + medication	Diabetic–plant-fish oil, slow carb diet
	NL	NF	DF	DFA	DPFS
Mean blood pressure (mm Hg)	107 ± 2	114 ± 7	103 ± 6	113 ± 2	100 ± 4
Systolic blood pressure (mm Hg)	135 ± 4	144 ± 7	126 ± 6	138 ± 3	127 ± 4
Diastolic blood pressure (mm Hg)	89 ± 3	91 ± 6	84 ± 6	96 ± 3	81 ± 4
Heart rate (beats per minute)	101 ± 7	109 ± 7	93 ± 11	102 ± 8	114 ± 4

Arterial pressures and heart rates (mean ± SEM) in normal (non-diabetic) and diabetic pigs are shown. There were no significant differences between the groups. The effects were measured 5 h after the start of the meal tolerance test.

### Hyperinsulinemic euglycemic clamps and 6,6-2H-glucose infusion

Fasting plasma metabolites and insulin are shown in **Table 4** (pre-clamp, week 6) and **Table 5** (clamp, week 6). **Table 4** shows that total cholesterol, HDL, LDL, VLDL, HDL/LDL and HDL/cholesterol differed among pig groups where the ratio between HDL and LDL or HDL and cholesterol were high (p<0.001) in NL and DFA pigs compared to the other groups. Non-esterified fatty acids (NEFA’s) were low (p<0.02) in the normal pigs and DPFS pigs, intermediate in the DFA pigs and high in the DF pigs. Beta-hydroxy-butyrate was below the detection limit of the assay in normal pigs and high (p<0.05) in DF pigs compared to normal pigs. Fructosamine and glucose (**Table 5**) were low (p<0.01) in the 2 groups of normal pigs compared to the 3 groups of D pigs. **Table 5** shows that insulin was high (p<0.01) in NF pigs compared to the D pig groups.

**Table 4:** Fasting plasma metabolite, hormone and enzyme concentrations prior the hyperinsulinemic, euglycemic clamp.

	Normal–Low fat, low sugar diet	Normal–Fast food	Diabetic–Fast food	Diabetic–Fast food + medication	Diabetic–Plant-Fish oil, Slow carb diet
	NL	NF	DF	DFA	DPFS
Cholesterol (mmol·L^-1^)	2^A^ ± 0	11^B^ ± 2	13^B^ ± 3	12^B^ ± 3	2^A^ ± 0
HDL (mmol·L^-1^)	0.8^AB^ ± 0.1	1.0^AB^ ± 0.1	1.6^C^ ± 0.2	1.3^BC^ ± 0.2	0.7^A^ ± 0.1
LDL (mmol·L^-1^)	1.2^A^ ± 0.1	5.0^B^ ± 0.6	5.3^B^ ± 0.8	5.6^B^ ± 0.8	0.9^A^ ± 0.2
VLDL (mmol·L^-1^)	0.1^A^ ± 0.0	4.9^B^ ± 1.2	6.6^B^ ± 1.4	5.0^B^ ± 1.7	0.1^A^ ± 0.0
HDL / LDL	0.74^B^ ± 0.05	0.21^A^ ± 0.02	0.30^A^ ± 0.03	0.25^A^ ± 0.03	0.82^B^ ± 0.12
HDL / cholesterol	0.41^B^ ± 0.02	0.10^A^ ± 0.01	0.14^A^ ± 0.02	0.13^A^ ± 0.02	0.42^B^ ± 0.04
Triglycerides (mmol·L^-1^)	0.29 ± 0.03	0.25 ± 0.02	0.58 ± 0.18	0.64 ± 0.26	0.34 ± 0.09
NEFA (mmol·L^-1^)	0.37^A^ ± 0.09	0.48^A^ ± 0.05	0.79^B^ ± 0.16	0.57^AB^ ± 0.12	0.48^A^ ± 0.19
B-OH-Butyrate (μmol· L^-1^)	<25^A^ ± 0	<25^A^ ± 0	196^B^ ± 94	84^AB^ ± 49	103^AB^ ± 73
Lactate (mmol·L^-1^)	0.7 ± 0.0	0.9 ± 0.1	1.0 ± 0.2	0.9 ± 0.1	0.9 ± 0.1
Fructosamine (μmol·L^-1^)	83^A^ ± 7	70^A^ ± 6	410^B^ ± 1.1	402^B^ ± 95	338^B^ ± 103
ALAT (μkat·L^-1^)	0.76^C^ ± 0.12	0.67^BC^ ± 0.11	0.43^A^ ± 0.08	0.49^AB^ ± 0.05	0.48^AB^ ± 0.06
ASAT (μkat·L^-1^)	<0.5	<0.5	<0.5	<0.5	<0.5
Cortisol (ng·ml^-1^)	60 ± 15	49 ± 10	89 ± 15	58 ± 15	55 ± 16
TNF*α* (pg·ml^-1^)	85^A^ ± 21	85^A^ ± 24	193^B^ ± 55	136^AB^ ± 40	107^A^ ± 31

Plasma concentrations (mean ± SEM) in normal (non-diabetic) and diabetic pigs are shown. Means sharing the same superscript A,B,C are not significantly different from each other.

HDL—high-density lipoprotein cholesterol; LDL—low density lipoprotein; VLDL—very low density lipoprotein; NEFA—non-esterified (“free”) fatty acids; ALAT—Alanine aminotransferase; ASAT—Aspartate aminotransferase; TNF*α*—tumor necrosis factor α.

**Table 5:** Parameters related to the hyperinsulinemic euglycemic clamp.

	Normal–Low fat, low sugar diet	Normal–Fast food	Diabetic–Fast food	Diabetic–Fast food + medication	Diabetic–Plant-Fish oil, Slow carb diet
	NL	NF	DF	DFA	DPFS
Body mass (kg)	117^B^ ± 3	126^B^ ± 5	98^A^ ± 6	107^AB^ ± 7	107^AB^ ± 5
**Pre-clamp/fasting parameters in plasma**					
Insulin (pmol·L^-1^)	33^BC^ ± 7	43^C^ ± 7	24^AB^ ± 4	26^AB^ ± 4	17^A^ ± 1
Glucose (mmol·L^-1^)	4.1^A^ ± 0.2	4.6^A^ ± 0.3	14.8^B^ ± 2.4	13.3^B^ ± 2.0	13.1^B^ ± 3.3
Hepatic glucose production (mg.kg^-1^.min^-1^)	2.3^A^ ± 0.1	2.8^AB^ ± 0.3	5.2^C^ ± 0.7	4.3^BC^ ± 0.5	4.9^C^ ± 1.2
**Steady state**					
Insulin (pmol·L^-1^)	419^C^ ± 14	407^BC^ ± 31	294^A^ ± 26	384^ABC^ ± 61	303^AB^ ± 33
Glucose (mmol·L^-1^)	4.4^A^ ± 0.1	4.2^A^ ± 0.2	5.2^B^ ± 0.3	4.5^A^ ± 0.1	4.7^A^ ± 0.1
Insulin-stimulated body glucose uptake (Rd) (mg.kg^-1^.min^-1^)	13.7 ± 2.0	12.3 ± 1.4	8.1 ± 2.2	9.5 ± 1.3	11.3 ± 2.7
Insulin-stimulated body glucose infusion rate (mg.kg^-1^.min^-1^)	13.9^B^ ± 1.4	11.6^AB^ ± 1.3	6.9^A^ ± 2.2	8.2^A^ ± 1.3	10.4^AB^ ± 2.7
Insulin-inhibited hepatic glucose production (mg.kg^-1^.min^-1^)	-0.17^A^ ± 0.76	0.76^AB^ ± 0.09	1.23^B^ ± 0.09	1.25^B^ ± 0.15	0.92^B^ ± 0.10

Body mass, plasma parameters (glucose and insulin concentrations) before (at the pre-clamp, fasting period) and at the steady state (at the end of hyperinsulinemic hyperglycemic clamps) and insulin-mediated whole body and hepatic glucose metabolism (mean ± SEM) in normal (non-diabetic) and diabetic pigs are shown. Means sharing the same superscript A,B,C are not significantly different from each other.

Inflammatory markers are shown in **Table 4** (pre-clamp, week 6). Plasma TNF-α was high (p<0.02) in DF pigs, intermediate in DFA pigs and low in DPFS pigs, the latter being comparable to both groups of normal pigs. Plasma cortisol was not different among pig groups. Plasma ALAT and ASAT were neither elevated in D compared to N pig groups nor between L and F.

Insulin-mediated glucose metabolism is shown in **Table 5** (clamp, week 6). Fasting hepatic glucose production was low (p<0.05) in the normal pigs and high in D pigs. Steady state plasma insulin concentrations during the clamp were similar among D pigs but DF and DPFS pigs showed low steady state plasma insulin concentrations compared to NL, indicating increased insulin clearance in DF and DPFS pigs at the standardized insulin-infusion rate of 2 mU.kg^-1^.min^-1^ during the clamp. Steady state plasma glucose concentrations during the clamp were ~10% higher in DF pigs compared to the other pig groups. This unwanted elevation and deviation from euglycemia during the clamp in DF pigs may cause an overestimation of insulin-stimulated whole-body glucose infusion rate in DF pigs due to the fact that plasma glucose itself can promote its own disposal through uptake by mass action into tissues and through suppression of hepatic glucose production [43, 44]. Insulin-inhibited hepatic glucose production was maximally supressed in NL pigs compared to the D pigs whereas NF pigs showed similar insulin-inhibited hepatic glucose production as the D pigs. Insulin-stimulated whole-body glucose infusion rate was high in NL pigs compared to both DF and DFA pigs. However, insulin-stimulated whole-body glucose infusion rate was similar among NL, NF and DPFS pigs. Coefficients of variation (CV%) of clamp plasma glucose concentrations, plasma insulin concentrations and glucose infusion rates during the steady state phase of the clamp were <11%, <14% and <6%, respectively except for plasma glucose and insulin concentrations in the NF pigs which were 16% and 31%, respectively.

### Necropsy

Postprandial plasma metabolite concentrations at 3 hours after initiation of the meal is shown in **Table 6** (pre-necropsy, week 7). Plasma glucose confirmed the observations from **Figs 3** and **4.** Fasting plasma NEFA’s were low (p<0.02) in the normal pigs and high in DF pigs compared to DFA and DPFS pigs. Postprandial plasma lactate was high (p<0.03) in DF and DFA pigs and low in DPFS pigs.

**Table 6:** Pre-prandial, post-prandial and response of plasma parameters prior to necropsy.

	Normal–Low fat, low sugar diet	Normal–Fast food	Diabetic–Fast food	Diabetic–Fast food + medication	Diabetic–Plant-Fish oil, Slow carb diet
	NL	NF	DF	DFA	DPFS
Glucose pre-prandial (mmol·L^-1^)	4.6^A^ ± 0.1	5.0^A^ ± 0.1	14^B^ ± 2	14^B^ ± 2	14^B^ ± 3
Glucose postprandial (mmol·L^-1^)	6.1^A^ ± 0.2	6.0^A^ ± 0.3	26^B^ ± 6	22^B^ ± 6	16^AB^ ± 4
Glucose response (mmol·L^-1^)	1.5^A^ ± 0.2	1.1^A^ ± 0.2	11^B^ ± 4	8^AB^ ± 4	2^A^ ± 2
NEFA pre-prandial (mmol·L^-1^)	0.21^A^ ± 0.07	0.19^A^ ± 0.02	0.48^B^ ± 0.10	0.36^AB^ ± 0.08	0.21^A^ ± 0.05
NEFA postprandial (mmol·L^-1^)	0.10^A^ ± 0.05	0.11^A^ ± 0.02	0.54^B^ ± 0.16	0.29^AB^ ± 0.10	0.26^AB^ ± 0.14
NEFA response (mmol·L^-1^)	-0.12 ± 0.03	-0.08 ± 0.02	0.06 ± 0.12	-0.07 ± 0.06	0.05 ± 0.10
B-OH-butyrate pre-prandial (μmol·L^-1^)	31 ± 7	<25 ± 0	146 ± 63	57 ± 23	49 ± 26
B-OH-butyrate postprandial (μmol·L^-1^)	26 ± 1	<25 ± 0	36 ± 8	<25 ± 0	73 ± 45
B-OH-butyrate response (μmol·L^-1^)	-5^B^ ± 1	0^B^ ± 7	-110^A^ ± 58	-32^AB^ ± 23	24^B^ ± 20
Lactate pre-prandial (mmol·L^-1^)	0.8 ± 0.1	1.4 ± 0.3	1.0 ± 0.1	1.1 ± 0.2	0.8 ± 0.1
Lactate postprandial (mmol·L^-1^)	1.5^AB^ ± 0.4	2.3^BC^ ± 0.2	3.2^C^ ± 0.6	2.7^C^ ± 0.3	0.9^A^ ± 0.2
Lactate response (mmol·L^-1^)	0.7^AB^ ± 0.2	0.9^ABC^ ± 0.5	2.2^C^ ± 0.6	1.6^BC^ ± 0.4	0.1^A^ ± 0.1

Plasma parameters^1)^ preceding (pre-prandial) and following (postprandial) a standardized meal size and the calculated response (postprandial minus pre-prandial) just prior necropsy (mean ± SEM) in normal (non-diabetic) and diabetic pigs are shown. Means sharing the same superscript A,B,C are not significantly different from each other.

^*1)*^ Blood samples were taken 15 minutes before the meal (pre-prandial) and 180 minutes after the meal (postprandial), just prior to necropsy and post-mortem examination and tissue collection.

**Fig 5:**
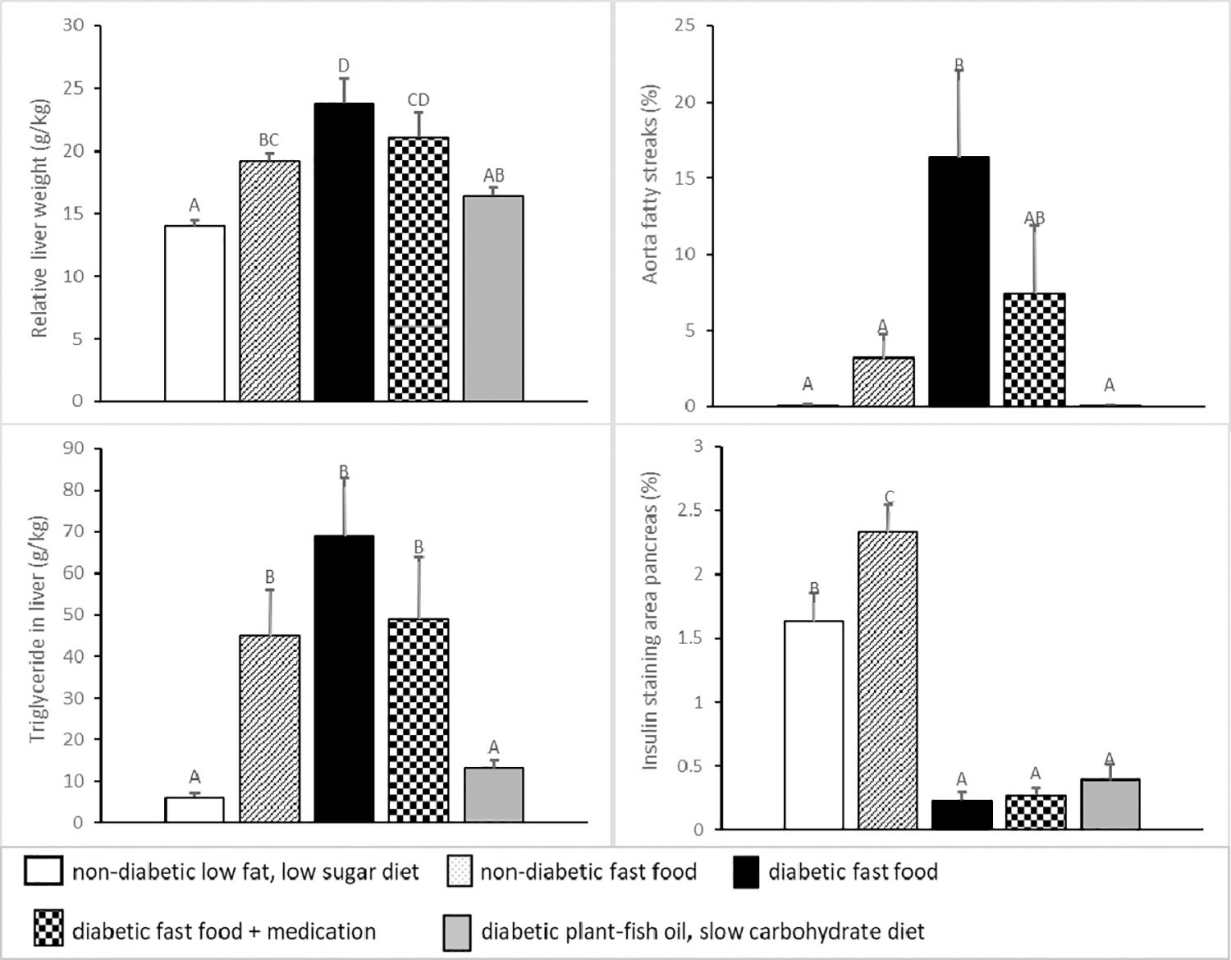
Body composition at scheduled necropsy (means±SEM) in five groups of normal (non-diabetic) and diabetic pigs fed a low fat, low sugar diet, fast food diet with or without medication or a plant-fish oil, slow carbohydrate diet. Means sharing the same superscript ABCD are not significantly different from each other.

Relative weights of organs and fat depots and body composition are shown in **Table 7** and **Fig 5**. Kidney weight (g.kg^-1^ bodyweight) was high (p<0.05) in DF pigs, intermediate in DFA and DPFS pigs, and low in NL and NF pigs (**Table 7**). Liver weight (g.kg^-1^ bodyweight) was high (p<0.05) in DF pigs, intermediate in DFA pigs and low in DPFS pigs, the latter being comparable to NL and NF pigs. However, NF showed high (p<0.05) liver weight compared to NL (**Fig 5**).

**Table 7:** Body mass and post-mortem relative weights of specific organs and tissues.

	Normal–Low fat, low sugar diet	Normal–Fast food	Diabetic–Fast food	Diabetic–Fast food + medication	Diabetic–plant-fish oil, slow carb diet
	NL	NF	DF	DFA	DPFS
Body mass at necropsy (kg)	122 ± 3^BC^	133 ± 5^C^	103 ± 7^A^	113 ± 8^AB^	114 ± 5^AB^
Kidney weight (g·kg^-1^)	2.6 ± 0.1^A^	2.4 ± 0.1^A^	3.8 ± 0.4^B^	3.2 ± 0.3^AB^	3.0 ± 0.3^AB^
Spleen weight (g·kg^-1^)	1.3 ± 0.1	1.5 ± 0.1	1.6 ± 0.1	1.4 ± 0.2	1.4 ± 0.1
Retroperitoneal fat weight (g·kg^-1^)	19.4 ± 1.9	23.5 ± 2.8	15.9 ± 2.3	16.8 ± 1.4	16.5 ± 2.7
Omental fat weight (g·kg^-1^)	1.9 ± 0.2	2.9 ± 0.4	2.3 ± 0.4	1.8 ± 0.2	1.9 ± 0.3
Mesenterial fat weight (g·kg^-1^)	7.7 ± 0.6	8.4 ± 0.7	7.5 ± 0.3	7.4 ± 0.6	6.5 ± 0.5
Thymus weight (g·kg^-1^)	0.38 ± 0.05	0.34 ± 0.03	0.30 ± 0.04	0.38 ± 0.04	0.37 ± 0.02

Results (mean ± SEM) in normal (non-diabetic) and diabetic pigs are shown. Means sharing the same superscript A,B,C are not significantly different from each other.

Triglyceride concentration in liver is shown in **Fig 5** and was high (p<0.05) in DF pigs, intermediate in DFA pigs and low in DPFS pigs, the latter being comparable to NL pigs. NF pigs showed high (p<0.05) liver triglyceride concentration compared to NL pigs. Triglyceride concentration in skeletal muscle (g.kg^-1^ muscle) was not different among groups: in NL, NF, DF, DFA and DPFS it was 10±4, 10±3, 11±3, 12±2 and 24±10, p = 0.1.

Atherosclerosis (aorta fatty streaks) is shown in **Fig 5**. In D pigs abdominal aorta fatty streak surface area (%) was high (p<0.05) in DF pigs, intermediate in DFA pigs and low in DPFS pigs. In normal pigs there was no significant difference in severity of atherosclerosis between L and F pigs.

Insulin staining of the pancreas is shown in **Fig 5**. Area of insulin staining (%) was high (p<0.05) in NF pigs compared to NL pigs. Area of staining was low in D pigs.

**Fig 6:**
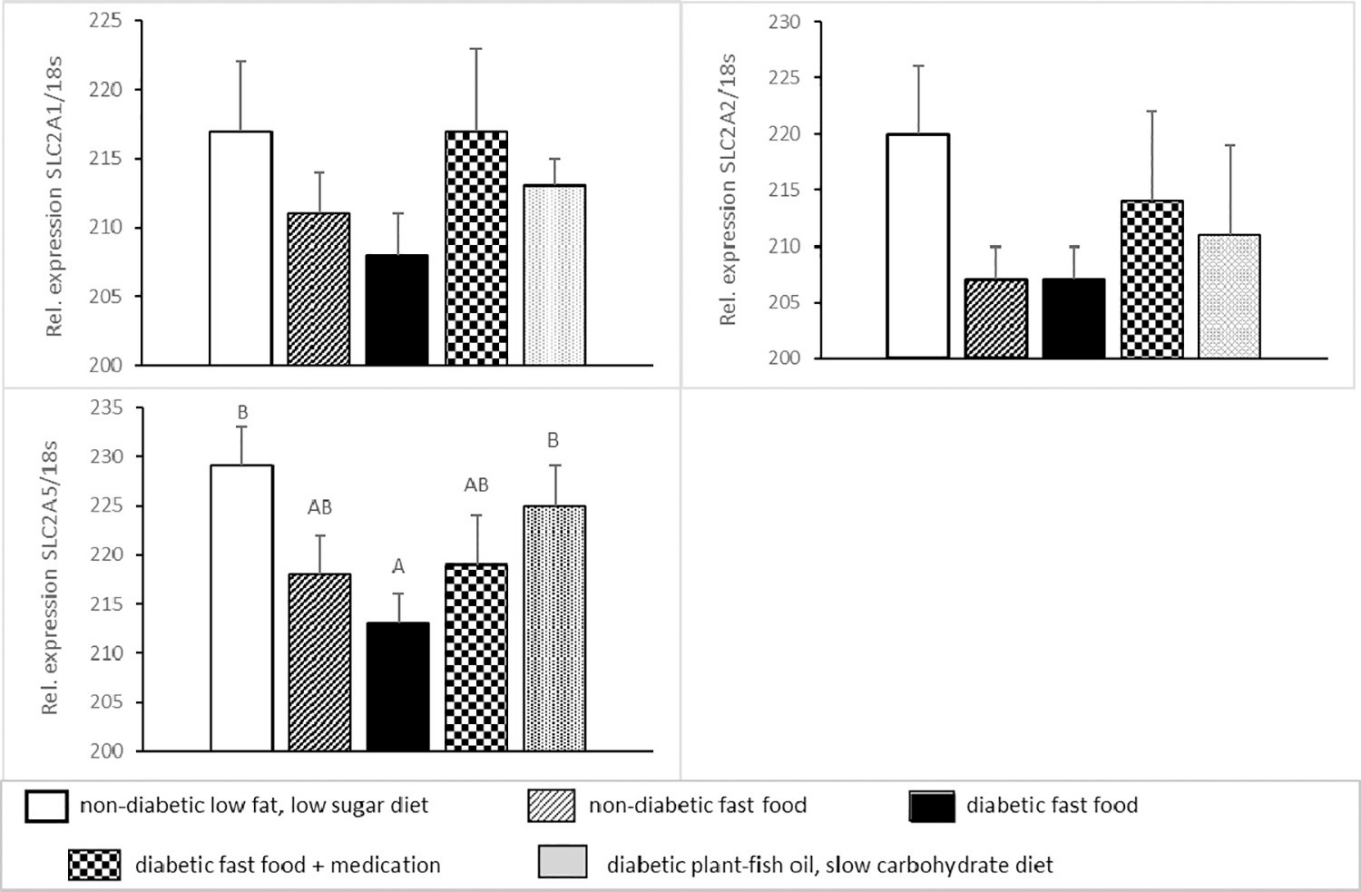
Relative glucose transporter SLC2A1, SLC2A2 and SLC2A5 gene expression corrected for 18S in the jejunal mucosa (means±SEM) in five groups of normal (non-diabetic) and diabetic pigs fed a low fat, low sugar diet, fast food diet with or without medication or a plant-fish oil, slow carbohydrate diet. Means sharing the same superscript A,B,C are not significantly different from each other.

Intestinal glucose transporters SLC2A1 (former GLUT1), SLC2A2 (former GLUT2) and SLC2A5 (former GLUT5) are shown in **Fig 6.** Relative expression of SLC2A5 was low (p<0.05) in DF pigs, intermediate in DFA pigs and high in DM pigs.

## Discussion

### Experimental design

This multifactorial study was primarily designed to compare the pathophysiology of 3 groups of diabetic pigs which each received a standardized isocaloric and isonitrogenous dietary treatment representing for what is considered an “unhealthy” or “healthy” dietary lifestyle for diabetic patients [5, 6]. We compared the cardiometabolic effects of an “unhealthy” Western dietary life style (i.e. a “fast” food type of diet, consisting of saturated fats, cholesterol and sugars) without or with taking oral anti-diabetic medication and a “healthy” plant-fish oil, slow carbohydrate dietary life style (i.e. “slow” food type of diet, consisting of unsaturated fats and slowly digestible starch). For reference, non-diabetic pigs were also studied to compare the severity of cardiometabolic effects of a “fast” food type of diet in subjects without and with diabetes. In the present study, pigs were studied until they were 7 months old, an age that is equivalent to human adolescence [26].

### Plant-fish oil, slow carbohydrate diet versus fast food diets without or with oral metformin-pioglitazone treatment

This study shows that a lifestyle adjustment, in the form of a plant-fish oil, slow carbohydrate diet compared to a fast food diet, improves cardiometabolic health in a type-2 like diabetes model in pigs. The major beneficial effects of a plant-fish oil, slow carbohydrate diet could be found in the field of inflammation (reduction of plasma TNF-α), early atherosclerosis (absence of fatty streaks in the abdominal aorta), reduced postprandial plasma glucose and triglyceride responses, and reduced postprandial plasma lactate concentrations indicative of improved glucose oxidation after a meal. Also fasting plasma NEFA concentrations were reduced to the level of normal pigs, suggesting a normalization of the balance between glucose and NEFA metabolism. Part of these improvements related to cardiometabolic health could also be observed in diabetic pigs on fast food with oral metformin-pioglitazone medication. However, the extent of improvement was less pronounced and intermediate between diabetic pigs on a fast food diet without oral medication and a plant-fish oil, slow carbohydrate diet. The beneficial effects of a plant-fish oil, slow carbohydrate diet may be partially related to the dietary unsaturated fats, especially originating from fish oil, which have affinity to bind to the nuclear peroxisome proliferator-activated receptor (PPAR), the same pathway which is activated by the anti-diabetic drug pioglitazone [3, 4, 45, 46]. Therefore, the plant-fish oil, slow carbohydrate diet may be regarded as a therapeutic means to prevent some of the abnormalities of type 2 diabetes. In this respect the Omega-3 fatty acids docosahexaenoic acid (DHA) and eicosapentaenoic acid (EPA) from fish oil have been shown to positively affect cardiovascular health, mainly through inhibition of inflammation [47, 48]. Lack of cholesterol and increased antioxidant potential of plant and fish oils in the diet may further reduce inflammation and early atherosclerosis [19, 49–52]. Slowly digestible starches in the plant-fish oil, slow carbohydrate diet may also help to reduce the postprandial plasma glucose and lactate responses, thereby improving meal-related glucose tolerance [53].

### Fast food versus low fat, low sugar diets

Fast food as compared to a low fat, low sugar diet in growing, non-diabetic Landrace pigs induced a limited number of cardiometabolic abnormalities related to metabolic syndrome. Full blown metabolic syndrome was not observed. It may be necessary to use adult, non-growing pigs of an obese breed to observe most of the abnormalities related to metabolic syndrome [24]. Superimposing streptozotocin-induced diabetes on fast food fed, growing Landrace pigs did induce severe cardiometabolic abnormalities like hyperglycemia, hypertriglyceridemia, hypo-insulinemia, increased plasma NEFA, lactate and TNF-α concentrations, high liver and kidney weights, high aorta fatty streak percentage and a low pancreatic beta cell mass, all related to the pathophysiology of obese type 2 diabetes in humans. Therefore, to properly mimic the cardiometabolic pathophysiology of human adolescent type 2 diabetes, the presently described streptozotocin-induced diabetic pig model which was fed a fast food instead of a low fat, low sugar [19, 20] diet is most valuable for translational research. These previous studies [19, 20] showed that low fat, low sugar but high carbohydrate (mainly digestible starches) diets had major adverse effects on glucose tolerance and insulin resistance but limited effects on lipid and NEFA metabolism and no effects on blood pressure and early atherosclerosis, thereby mimicking only part of the cardiometabolic abnormalities of obese type 2 diabetes in humans.

### Food intake and body weight gain

We administered the pigs 2 meals per day and the pigs were allowed to eat ad libitum for a period of one hour. In this way we could quantify the possible appetizing or satiating effects of metformin-pioglitazone treatment or a plant-fish oil, slow carbohydrate diet compared to a fast food diet. For translational purposes this mimics the day to day situation of human diabetic type 2 patients best. On the other hand, any differences in ad libitum food intake and body weight gain between diabetic pig groups may obscure the interpretation of the direct effects of metformin-pioglitazone and/or plant-fish oil, slow carbohydrate diet on cardiometabolic health. Both metformin and pioglitazone have been reported to influence appetite and food intake: metformin having neutral to reducing effects on food intake [54] whereas pioglitazone has been shown to increase food intake [55]. The possible appetizing or satiating effects of a plant-fish oil, slow carbohydrate diet in (untreated) diabetic subjects is to the best of our knowledge unknown. Increased food intake and body weight gain reduce cardiometabolic health and insulin sensitivity and vice versa [56]. In our study, the net effect of metformin-pioglitazone treatment or a plant-fish oil, slow carbohydrate diet on ad libitum food intake and body weight gain in diabetic pigs was positive: a (non-significant) increase in food intake of ~10% was registered compared to diabetic pigs on a fast food without medication. This 10% increase in food intake and body weight gain may mask part of the beneficial cardiometabolic and insulin sensitizing effects of metformin-pioglitazone and the plant-fish oil, slow carbohydrate diet. As a consequence, the beneficial effects of the metformin-pioglitazone treatment and the plant-fish oil, slow carbohydrate diet compared to the fast food diet without medication may be somewhat underestimated in the present study. The comparison between metformin-pioglitazone treatment and plant-fish oil, slow carbohydrate diet is however not influenced by differences in caloric intake and body weight gain.

### Glucose metabolism

The mechanisms behind the improved postprandial glucose tolerance in DPFS pigs compared to DF pigs seem related to various significant factors and trends. All these factors combined may add up and result in better metabolic health. Significant and numerical improvements in fasting NEFA, B-hydroxy-butyric acid, TNF-α, cortisol and postprandial triglycerides, lactate and insulin-mediated whole-body glucose metabolism all contribute to improved glucose tolerance in DPFS pigs.

SLC2A1, SLC2A2 and SLC2A5 gene expression in mid-jejunal mucosa was measured to investigate whether intestinal glucose transporters could be influenced by diabetes, diet and/or metformin-pioglitazone medication and as such have an effect on the postprandial plasma glucose concentrations. SLC2A1 and SLC2A2 were not affected among pig groups but SLC2A5 showed a modest reduction in DF pigs whereas DPFS pigs showed similar levels of SLC2A5 as compared to the normal pigs. From these results it can be concluded that SLC2A5 gene expression in jejunal mucosa, being the small intestinal location where glucose absorption from food is the highest [25], has no major impact on postprandial glucose tolerance. Our results on SLC2A1,2,5 gene expression are in contrast to previous work in diabetic rats and humans where an increase in SLC2A1,2,5 gene expression was found [57, 58]. Reasons for this discrepancy may lay in the fact that in our study intestinal SLC2A2 expression was measured 3-h postprandially at high intestinal nutrient fluxes (representative for meal glucose tolerance) whereas in the other studies intestinal SLC2A2 expression was measured in the fasting state at low intestinal nutrient fluxes. Furthermore, location in the small intestine as well as species and diet differences may all affect intestinal SLC2A2 expression [57, 58].

### Early atherosclerosis and blood pressure

DPFS, NL and NF pigs had no to little fatty streaks in the abdominal aorta which is mainly related to the absence of cholesterol in the plant-fish oil, slow carbohydrate and low fat, low sugar diets [19, 59] and for the NF pigs the relatively short (7 weeks) exposure to the F diet. However, within the same 7 week time-frame, D pigs do develop fatty streaks in the abdominal aorta on the F diet and D pigs are therefore more sensitive to develop early atherosclerosis than N pigs, which confirms previous research [36]. DFA pigs showed intermediate early atherosclerosis compared to DF and DPFS pigs which indicates that combined metformin and pioglitazone therapy has inhibiting effects on the development of atherosclerosis in diabetic pigs, a phenomenon which has also been observed in humans [46, 60]. Blood pressure was not significantly different among pig groups, which may be related to the fact that blood pressure mainly correlates with body weight and visceral body fat composition [61, 62], which was, in the present study, similar for retroperitoneal, omental and mesenterial fat mass.

### Plant-fish oil, slow carbohydrate-type diets studied in obese or diabetic human patients

Association between eating behavior (lifestyle and fast food), obesity and occurrence of chronic cardio-metabolic diseases (such as type 2 diabetes mellitus) in Western societies gains increasing attention of experts world-wide [5, 6, 12]. The associations between diverse nutritional factors (such as daily intakes of specific sources of fats and fatty acids, i.e., saturated, monounsaturated and polyunsaturated–SFA, MUFA and PUFA) and the occurrence of some comorbidities such as type 2 diabetes mellitus and cardio-vascular diseases have been examined by a number of national or international institutions (https://ec.europa.eu/jrc/en/health-knowledge-gateway/promotion-prevention/nutrition/fats), which indicated that replacing SFA with PUFA, and in some cases MUFA, has beneficial effects on cardiometabolic clinical endpoints, while all agree that replacing total dietary fatty acids with PUFA and MUFA has the most favorable effects on serum lipid concentration and coronary heart disease risk. Indeed, adherence to a plant-fish oil, slow carbohydrate-type diet was associated with improved control of cardiovascular risk factors in obese type 2 diabetic patients with poor control [15]. More specifically, it has been described that supplementation of MUFAs and PUFAs (besides the differences in their structures and properties) have different effects on chronic diseases [17, 18]. These studies indicated that the metabolic phenotype of subjects clearly determines response to the quantity and quality of dietary fat on cardiometabolic risk factors, which suggests that targeted and personalized dietary therapies may be of value for its different metabolic features. In other words, each specific chronic disease may require its own tailor-made MUFA and PUFA enriched diet for maximum beneficial effect on cardiometabolic health.

## Conclusion

Fast food predisposes to the development of metabolic syndrome in young growing non-diabetic subjects and the diabetic condition amplifies the adverse effects of fast food on cardiometabolic health. This translational study in diabetic pigs shows that the beneficial effects of a plant-fish oil, slow carbohydrate diet on cardiometabolic health exceed the correcting effects of oral anti-diabetic medication while maintaining a fast-food diet.

## Supporting information

S1 Data Individual data of normal (non-diabetic) and diabetic pigs. (XLS)

## Abbreviations

AUC: area under the curve
DF: diabetic pigs on fast food diet
DFA: diabetic pigs on fast food diet with anti-diabetic medication (oral metformin-pioglizatone)
DPFS: diabetic pigs on a plant-fish oil, slow carbohydrate diet
ME: metabolizable energy
NEFA: non-esterified fatty acids
NF: normal pigs on fast food diet
NL: normal pigs on low fat, low sugar pig diet
PPAR: peroxisome proliferator-activated receptor
SLC2A: glucose transporter
STZ: streptozotocin
